# Infection prevalence of *Sodalis* symbionts among stinkbugs

**DOI:** 10.1186/s40851-014-0009-5

**Published:** 2015-01-28

**Authors:** Takahiro Hosokawa, Nahomi Kaiwa, Yu Matsuura, Yoshitomo Kikuchi, Takema Fukatsu

**Affiliations:** Bioproduction Research Institute, National Institute of Advanced Industrial Science and Technology (AIST), Tsukuba, 305-8566 Japan; Tropical Biosphere Research Center, University of the Ryukyus, Okinawa, 903-0213 Japan; Department of General Systems Studies, Graduate School of Arts and Science, University of Tokyo, Tokyo, 153-8902 Japan; Graduate School of Environmental Science, Hokkaido University, Sapporo, 060-0810 Japan; Bioproduction Research Institute, Hokkaido Center, National Institute of Advanced Industrial Science and Technology (AIST), Sapporo, 062-8517 Japan; Graduate School of Life and Environmental Sciences, University of Tsukuba, Tsukuba, 305-8572 Japan; Department of Biological Sciences, University of Tokyo, Tokyo, 113-0033 Japan

**Keywords:** *Sodalis*, Hemiptera, Heteroptera, Stinkbug, Facultative symbiont, Infection frequency, Natural population

## Abstract

**Introduction:**

Diverse insects and other organisms are associated with microbial symbionts, which often significantly contribute to growth and survival of their hosts and/or drastically affect phenotypes of their hosts in a variety of ways. *Sodalis glossinidius* was first identified as a facultative bacterial symbiont of tsetse flies, and recent studies revealed that *Sodalis-*allied bacteria encompass diverse ecological niches ranging from free-living bacteria through facultative symbionts to obligate symbionts associated with a diverse array of insects. Despite potential ecological and evolutionary relevance of the *Sodalis* symbionts, their infection prevalence in natural insect populations has been poorly investigated.

**Results:**

Here we surveyed diverse stinkbugs and allied terrestrial heteropteran bugs, which represented 17 families, 77 genera, 108 species, 310 populations and 960 individuals, for infection with the *Sodalis* symbionts. Diagnostic PCR detected relatively low infection frequencies of the *Sodalis* symbionts: 13.6% (14/103) of the species, 7.5% (22/295) of the populations, and 4.3% (35/822) of the individuals of the stinkbugs except for those belonging to the family Urostylididae. Among the urostylidid stinkbugs, strikingly, the *Sodalis* symbionts exhibited very high infection frequencies: 100% (5/5) of the species, 100% (15/15) of the populations, and 94.2% (130/138) of the individuals we examined. Molecular phylogenetic analysis based on bacterial 16S rRNA gene sequences revealed that all the symbionts were placed in the clade of *Sodalis*-allied bacteria while the symbiont phylogeny did not reflect the systematics of their stinkbug hosts. Notably, the *Sodalis* symbionts of the urostylidid stinkbugs were not clustered with the *Sodalis* symbionts of the other stinkbug groups on the phylogeny, suggesting their distinct evolutionary trajectories.

**Conclusions:**

The relatively low infection frequency and the overall host-symbiont phylogenetic incongruence suggest that the *Sodalis* symbionts are, in general, facultative symbiotic associates in the majority of the stinkbug groups. On the other hand, it is conceivable, although speculative, that the *Sodalis* symbionts may play some substantial biological roles for their host stinkbugs of the Urostylididae.

**Electronic supplementary material:**

The online version of this article (doi:10.1186/s40851-014-0009-5) contains supplementary material, which is available to authorized users.

## Introduction

Diverse insects are associated with symbiotic microorganisms [1]. Some symbionts are obligate companions essential for their hosts via, for example, provisioning of essential nutrients deficient in their host’s diets, and often referred to as the primary symbionts [2,3]. Other symbionts are facultative associates not essential for their hosts, and often designated as the secondary symbionts [4,5]. Although not needed for their host’s survival, many of the facultative symbionts drastically affect various adaptive phenotypes of their hosts, which include manipulating host’s reproductive phenotypes in selfish ways [6,7], conferring host’s resistance to parasites and pathogens [8-10], enhancing host’s tolerance to heat stress [11,12], broadening host’s food plant range [13,14], modifying host’s body color [15,16] and others.

Grasping infection prevalence of these symbionts is important for gaining insights into biological interactions with their hosts. The primary symbionts of obligate nature generally exhibit 100% infection frequencies in their host populations due to their indispensable roles. By contrast, the secondary symbionts of facultative nature exhibit variable infection frequencies ranging from near 0% to almost 100% depending on the symbiont species, the host species and populations, the environmental conditions, etc. For example, some *Wolbachia* strains attain 100% infection frequencies in their host populations by their selfish driving mechanisms such as cytoplasmic incompatibility and parthenogenesis induction [6,7]. The facultative symbionts *Serratia*, *Regiella* and *Hamiltonella* in natural aphid populations exhibit intermediate values between 0% to 100% [17-20], which probably reflect their context-dependent fitness consequences [8,9,11,13].

*Sodalis glossinidius* was first identified and described as a gammaproteobacterial secondary symbiont of tsetse flies (Diptera: Glossinidae) [21-23]. Subsequently, primary symbionts associated with bacteriocytes of *Sitophilus* grain weevils (Coleoptera: Curculionidae) turned out to be closely related to *Sodalis* [24,25] and designated as ‘*Candidatus* Sodalis pierantonius’ [26]. Recently, a number of studies have reported occurrences of *Sodalis*-allied bacteria in a diverse array of insects: as bacteriocyte-associated presumable primary symbionts in bird lice (Phthiraptera: Philopteridae) [27,28], louse flies (Diptera: Hippoboscidae) [29,30], spittle bugs (Hemiptera: Cercopoidea) [31,32] and pseudococcids (Hemiptera: Pseudococcidae) [33]; and as presumable secondary symbionts in acorn weevils (Coleoptera: Curculionidae) [34-36], longicorn beetles (Coleoptera: Cerambycidae) [37], stinkbugs (Hemiptera: Scutelleridae and Pentatomidae) [38-41], psyllids (Hemiptera: Triozidae) [42] and archaeococcoid scale insects (Hemiptera: Coelostomidiidae) [43]. Furthermore, a *Sodalis*-allied bacterial strain was isolated from a human wound infection [44], and a biofilm-forming bacterium isolated from a tufa deposit, *Biostraticola tofi*, turned out to be a close relative of *Sodalis* [45], uncovering diverse ecological niches and symbiotic statuses of the *Sodalis*-allied bacteria. While infection frequencies in natural insect populations have been extensively surveyed for *Wolbachia* [46-48], *Rickettsia* [49,50], *Cardinium* [51-53], *Spiroplasma* [52,54], *Arsenophonus* [52,55] and other facultative symbionts, no systematic and comprehensive survey of *Sodalis* symbionts in natural host populations has been reported.

In this study, we surveyed diverse stinkbugs and allied terrestrial heteropteran bugs (order Hemiptera: suborder Heteroptera: infraorder Pentatomomorpha), which represent 17 families, 77 genera, 108 species, 310 populations and 960 individuals, for infection with *Sodalis* symbionts by diagnostic PCR and molecular phylogenetic approaches.

## Materials and methods

### Insect samples

Additional file 1 lists the insect samples examined in this study. These insects were preserved in either acetone or ethanol [56], or freshly brought to the laboratory. For large specimens, dissected gonad was subjected to DNA extraction. For small specimens, dissected abdomen was subjected to DNA extraction. DNA extraction was performed using QIAamp DNA Mini kit (Qiagen).

### PCR, cloning and sequencing

A 1.5 kb region of the bacterial 16S rRNA gene was amplified by PCR with primers 16SA1 (5’-AGA GTT TGA TCM TGG CTC AG-3’) and 16SB1 (5’-TAC GGY TAC CTT GTT ACG ACT T-3’), and cloned and sequenced as described previously [57]. Diagnostic PCR was performed with primers sodalis370F (5’-CGR TRG CGT TAA YAG CGC-3’) [38] and 16SB1 under the temperature profile of 95°C for 10 min followed by 35 cycles of 94°C for 30 sec, 55°C for 1 min and 72°C for 1.5 min. For quality control of the DNA samples, a 1.5 kb region of mitochondrial 16S rRNA gene was amplified by PCR with primers MtrA1 (5’-AAW AAA CTA GGA TTA GAT ACC CTA-3’) and MtrB1 (5’-TCT TAA TYC AAC ATC GAG GTC GCA A-3') [58].

### Molecular phylogenetic analysis

A multiple alignment of the nucleotide sequences was generated by the program MAFFT version 7.127b [59]. The nucleotide substitution model, GTR + I + G, was selected using the program jModelTest 2 [60,61]. The phylogenetic analyses were conducted by Bayesian (BA) and maximum-likelihood (ML) methods with the programs MrBayes v3.2.2 [62] and RAxML version 7.2.6 [63], respectively. In the BA analysis, in total 37,500 trees were obtained for each analysis (ngen = 50,000,000, samplefreq = 1,000, burn in = 12,501, temp = 0.2) and multiple independent runs were conducted to ensure the stable results. Posterior probabilities were calculated for each node by statistical evaluation in BA, whereas bootstrap values were obtained with 1000 replications in ML.

## Results and discussion

Our diagnostic PCR survey of diverse stinkbugs and allied terrestrial heteropteran bugs, which represent 17 families, 77 genera, 108 species, 310 populations and 960 individuals, detected *Sodalis* symbionts from 17.6% (19/108) of the species, 11.0% (34/310) of the populations, and 17.2% (165/960) of the individuals (Table 1; Additional file 1). The infection frequencies were generally low and considerably variable among the different heteropteran groups: at the individual level, for example, 15.4% (4/26) in the Acanthosomatidae; 0.0% (0/33) in the Cydnidae; 5.6% (18/323) in the Pentatomidae; 0.0% (0/40) in the Plataspidae; 4.5% (11/247) in the Scutelleridae; 0.0% (0/30) in the Alydidae; 0.0% (0/45) in the Coreidae; and 0.0% (0/43) in the Blissidae (Table 1; Additional file 1). In the Urostylididae, by contrast, infection frequencies with the *Sodalis* symbionts were exceptionally high: 100% (5/5) of the species, 100% (15/15) of the populations and 94.2% (130/138) of the individuals (Table 1; Additional file 1) [40].

**Table 1:** **Detection of** ***Sodalis*** **symbionts from stinkbugs representing 17 families, 77 genera, 108 species, 310 populations and 960 individuals collected in Japan**

**Superfamily family**	**Genus**	**Species**	**Population**	**Individual**
Pentatomoidea				
Acanthosomatidae	1/4 (25.0%)	1/4 (25.0%)	2/10 (20.0%)	4/26 (15.4%)
Cydnidae	0/3 (0.0%)	0/5 (0.0%)	0/8 (0.0%)	0/33 (0.0%)
Dinidoridae	0/1 (0.0%)	0/1 (0.0%)	0/5 (0.0%)	0/12 (0.0%)
Parastrachiidae	0/1 (0.0%)	0/1 (0.0%)	0/1 (0.0%)	0/10 (0.0%)
Pentatomidae	8/37 (21.6%)	8/51 (15.7%)	15/189 (7.9%)	18/323 (5.6%)
Platasipidae	0/3 (0.0%)	0/8 (0.0%)	0/16 (0.0%)	0/40 (0.0%)
Scutelleridae	3/7 (42.9%)	3/8 (37.5%)	3/29 (10.3%)	11/247 (4.5%)
Urostylididae	2/2 (100%)	5/5 (100%)	15/15 (100%)	130/138 (94.2%)
Coreoidea				
Alydidae	0/1 (0.0%)	0/1 (0.0%)	0/1 (0.0%)	0/30 (0.0%)
Coreidae	0/9 (0.0%)	0/13 (0.0%)	0/19 (0.0%)	0/45 (0.0%)
Rhopalidae	1/1 (100%)	1/1 (100%)	1/1 (100%)	1/1 (100%)
Lygaeoidea				
Berytidae	0/1 (0.0%)	0/1 (0.0%)	0/2 (0.0%)	0/3 (0.0%)
Blissidae	0/1 (0.0%)	0/1 (0.0%)	0/5 (0.0%)	0/43 (0.0%)
Lygaeidae	0/1 (0.0%)	0/1 (0.0%)	0/1 (0.0%)	0/1 (0.0%)
Rhyparochromidae	0/3 (0.0%)	0/3 (0.0%)	0/3 (0.0%)	0/3 (0.0%)
Pyrrhocoroidea				
Largidae	0/1 (0.0%)	0/2 (0.0%)	0/3 (0.0%)	0/3 (0.0%)
Pyrrhocoridae	1/1 (100%)	1/2 (50.0%)	1/2 (50.0%)	1/2 (50.0%)
Total	16/77 (20.8%)	19/108 (17.6%)	34/310 (11.0%)	165/960(17.2%)
Total without Urostylididae	14/75 (18.7%)	14/103 (13.6%)	22/295 (7.5%)	35/822 (4.3%)

**Figure 1:**
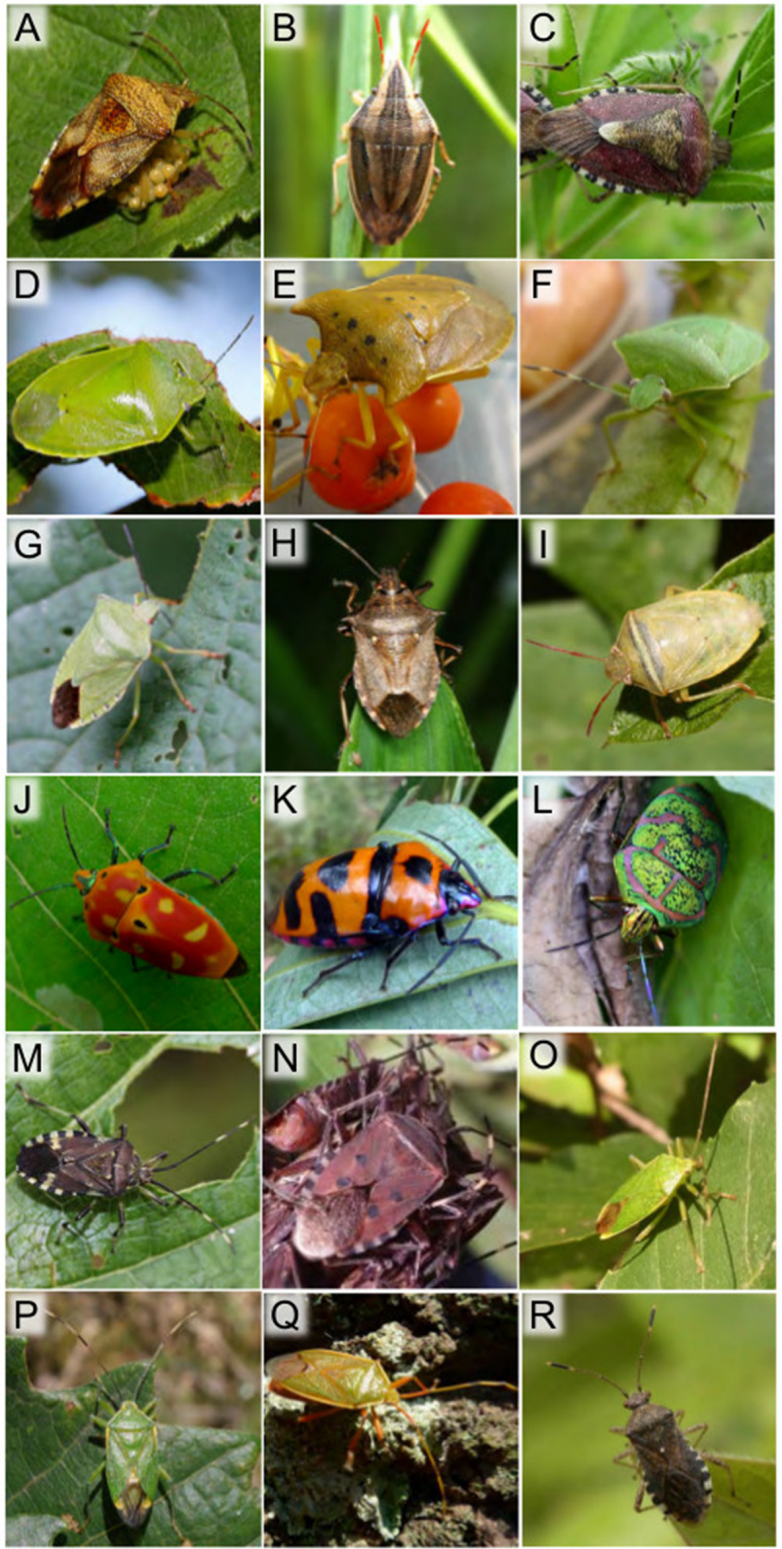
**Stinkbugs associated with the** ***Sodalis*** **symbionts. (A)** *Elasmucha putoni*. **(B)** *Aelia fieberi*. **(C)** *Dolycoris baccarum*. **(D)** *Glaucias subpunctatus*. **(E)** *Lelia decempunctata*. **(F)** *Nezara antennata*. **(G)** *Palomena angulosa*. **(H)** *Picromerus lewisi*. **(I)** *Piezodorus hybneri*. **(J)** *Cantao ocellatus*. **(K)** *Eucorysses grandis*. **(L)** *Poecilocoris lewisi*. **(M)** *Urochela luteovaria*. **(N)** *Urochela quadrinotata*. **(O)** *Urostylis annulicornis*. **(P)** *Urostylis striicornis*. **(Q)** *Urostylis westwoodii*. **(R)** *Rhopalus sapporensis*. Photos by Toru Kawabe **(A-D, G, I, L, M and** **R)**, Takahiro Hosokawa **(E, F, J, K and** **Q)**, Joji Yokozeki **(H)**, Gaku Miyake **(N)**, Nahomi Kaiwa **(O)** and Yoshishige Shinogi **(P)**.

**Figure 2:**
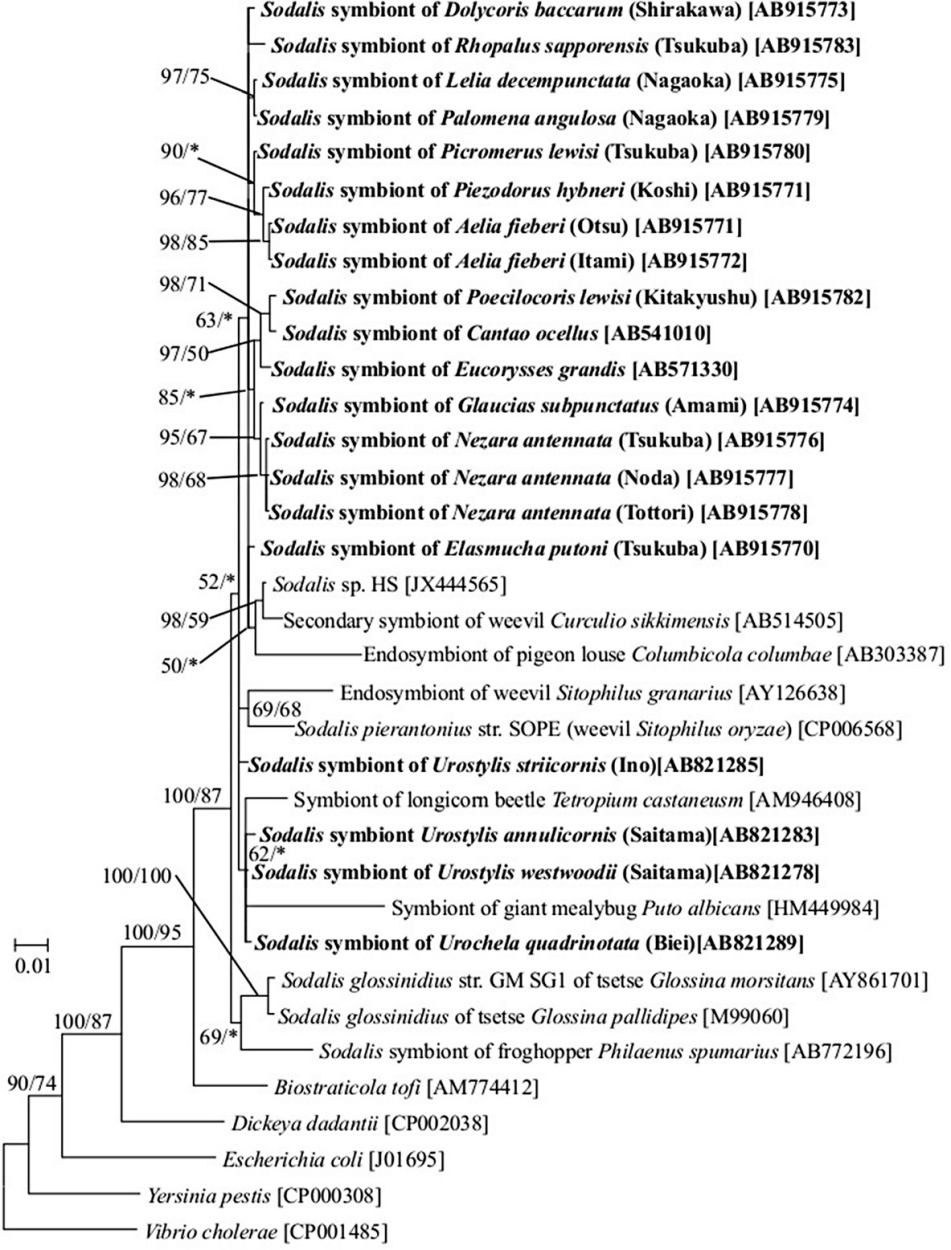
**Phylogenetic relationship between** ***Sodalis*** **symbionts of heteropteran bugs and other insects inferred from 16S rRNA gene sequences (1204 aligned nucleotide sites).** A Bayesian phylogeny is shown with statistical support values (50% or higher) at the nodes: posterior probabilities of Bayesian analysis/bootstrap probabilities of maximum likelihood analysis. Asterisks indicate support values lower than 50%. Sequences obtained from stinkbugs are highlighted by boldface, wherein collection localities are indicated in parentheses and accession numbers in brackets.

In previous studies, 16S rRNA gene sequences of the *Sodalis* symbionts were determined for two scutellerid species *Cantao ocellatus* and *Eucoryses grandis* [38,39] and four urostylidid species *Urostylis annulicornis*, *Urostylis striicornis*, *Urostylis westwoodii* and *Urochela quadrinotata* [40]. In this study, we newly cloned and sequenced 16S rRNA gene of the *Sodalis* symbionts from the following heteropteran species: an acanthosomatid *Elasmucha putoni*; pentatomids *Aelia fieberi* (from two populations), *Dolycoris baccarum*, *Glaucias subpunctatus*, *Lelia decempunctata*, *Nezara antennata* (from three populations), *Palomena angulosa*, *Picromerus lewisi* and *Piezodorus hybneri*; a scutellerid *Poecilocoris lewisi*; and a rhopalid *Rhopalus sapporensis* (Figure 1). Molecular phylogenetic relationship of the *Sodalis* symbionts associated with the heteropteran bugs and other insects was inferred from the 16S rRNA gene sequences (Figure 2). The phylogenetic pattern indicated that (i) all the symbiont sequences were placed in the clade of *Sodalis*-allied bacteria with high statistical supports, (ii) the symbiont sequences within the same host species tended to be closely related to each other, (iii) nonetheless, the overall phylogenetic relationship of the symbiont sequences did not reflect the systematics of the host stinkbugs, and (iv) notably, the *Sodalis* symbionts of the urostylidid stinkbugs were not clustered with the *Sodalis* symbionts of the other stinkbug groups on the phylogeny.

The relatively low infection frequencies and the overall host-symbiont phylogenetic incongruence favor the hypothesis that the *Sodalis* symbionts are, in general, facultative associates for the heteropteran bugs, as *Wolbachia*, *Rickettsia*, *Spiroplasma*, *Lariskella*, etc. [41,64-66]. The majority of the plant-sucking heteropteran bugs harbor specific gut bacteria as the primary symbionts within the crypt cavities present in a posterior midgut region [1,67,68], which are important for normal growth, survival and reproduction of the host insects [69-80]. Probably, the majority of the *Sodalis* symbionts are, unlike the primary gut symbionts, not essential for their heteropteran hosts. On the other hand, it is conceivable, although speculative, that the *Sodalis* symbionts may play some substantial biological roles for their host stinkbugs in the Urostylididae. It deserves future studies what biological roles, which are likely condition-dependent ones, the *Sodalis* symbionts play for their urostylidid hosts.

## Conclusions

In conclusion, our results highlight that the *Sodalis* symbionts are facultative symbiotic bacteria commonly associated with diverse insects, as are *Wolbachia*, *Rickettsia*, *Spiroplasma*, *Cardinium*, *Arsenophonus* and other widespread facultative symbionts. In this study, we exhaustively surveyed diverse stinkbugs in Japan, but, considering the recent report on the infection prevalence of the *Sodalis* symbiont in African populations of the coffee bug *Antestiopsis thunbergii* (Pentatomidae) [41], the occurrence of the *Sodalis* symbionts seems widespread among world’s stinkbugs and other insects. Future studies should focus on comprehensive survey of insect groups other than the heteropteran bugs, and also on effects and consequences of their infection to the host insects. Comparative studies on *Sodalis*-infected and uninfected host insects under the same genetic background combined with genomic and molecular biological analyses of the *Sodalis* symbionts will provide insights into ecological and evolutionary aspects of animal-microbe symbioses wherein the associations may range from free-living through facultative to obligate.

## Additional file

Additional file 1:
**Stinkbug samples examined in this study and detection of** ***Sodalis*** **symbionts from the samples.**

## References

[1] Buchner P (1965). Endosymbiosis of animals with plant microorganisms.

[2] Moran NA, McCutcheon JP, Nakabachi A (2008). Genomics and evolution of heritable bacterial symbionts. Annu Rev Genet.

[3] Douglas AE (2009). The microbial dimension in insect nutritional ecology. Funct Ecol.

[4] Oliver KM, Degnan PH, Burke GR, Moran NA (2010). Facultative symbionts in aphids and the horizontal transfer of ecologically important traits. Annu Rev Entomol.

[5] Feldharr H (2010). Bacterial symbionts as mediators of ecologically important traits of insect hosts. Ecol Entomol.

[6] O’Neill SL, Hoffmann AA, Werren JH (1997). Influential passengers: inherited microorganisms and arthropod reproduction.

[7] Werren JH, Baldo L, Clark ME (2008). *Wolbachia*: master manipulators of invertebrate biology. Nat Rev Microbiol.

[8] Oliver KM, Russell JA, Moran NA, Hunter MS (2003). Facultative bacterial symbionts in aphids confer resistance to parasitic wasps. Proc Natl Acad Sci U S A.

[9] Scarborough CL, Ferrari J, Godfray HCJ (2005). Aphid protected from pathogen by endosymbiont. Science.

[10] Hedges LM, Brownlie JC, O’Neill SL, Johnson KN (2008). *Wolbachia* and virus protection in insects. Science.

[11] Montllor CB, Maxmen A, Purcell AH (2002). Facultative bacterial endosymbionts benefit pea aphids *Acyrthosiphon pisum* under heat stress. Ecol Entomol.

[12] Russell JA, Moran NA (2006). Costs and benefits of symbiont infection in aphids: variation among symbionts and across temperatures. Proc R Soc B.

[13] Tsuchida T, Koga R, Fukatsu T (2004). Host plant specialization governed by facultative symbiont. Science.

[14] Tsuchida T, Koga R, Matsumoto S, Fukatsu T (2011). Interspecific symbiont transfection confers a novel ecological trait to the recipient insect. Biol Let.

[15] Tsuchida T, Koga R, Horikawa M, Tsunoda T, Maoka T, Matsumoto S, Simon JC, Fukatsu T (2010). Symbiotic bacterium modifies aphid body color. Science.

[16] Tsuchida T, Koga R, Fujiwara A, Fukatsu T (2014). Phenotypic effect of “*Candidatus* Rickettsiella viridis”, a facultative symbiont of the pea aphid (*Acyrthosiphon pisum*), and its interaction with a coexisting symbiont. Appl Environ Microbiol.

[17] Tsuchida T, Koga R, Shibao H, Matsumoto T, Fukatsu T (2002). Diversity and geographic distribution of secondary endosymbiotic bacteria in natural populations of the pea aphid, *Acyrthosiphon pisum*. Mol Ecol.

[18] Simon JC, Boutin CM, Prunier–Leterme N, Sabater–Muñoz B, Latorre A, Bournoville R (2003). Host–based divergence in populations of the pea aphid: insights from nuclear markers and the prevalence of facultative symbionts. Proc R Soc B.

[19] Russell JA, Latorre A, Sabater-Muñoz B, Moya A, Moran NA (2003). Side-stepping secondary symbionts: widespread horizontal transfer across and beyond the Aphidoidea. Mol Ecol.

[20] Haynes S, Darby AC, Daniell TJ, Webster G, van Veen FJF, Godfray HCJ, Prosser JI, Douglas AE (2003). Diversity of bacteria associated with natural aphid populations. Appl Environ Microbiol.

[21] Beard CB, O’Neill SL, Mason P, Mandelco L, Woese CR, Tesh RB, Richards FF, Aksoy S (1993). Genetic transformation and phylogeny of bacterial symbionts from tsetse. Insect Mol Biol.

[22] Aksoy S, Chen X, Hypsa V (1997). Phylogeny and potential transmission routes of midgut-associated endosymbionts of tsetse (Diptera: Glossinidae). Insect Mol Biol.

[23] Dale C, Maudlin I (1999). *Sodalis* gen. nov. and *Sodalis glossinidius* sp. nov., a microaerophilic secondary endosymbiont of the tsetse fly *Glossina morsitans morsitans*. Int J Syst Evol Microbiol.

[24] Heddi A, Grenier AM, Khatchadourian C, Charles H, Nardon P (1999). Four intracellular genomes direct weevil biology: nuclear, mitochondrial, principal endosymbiont, and *Wolbachia*. Proc Natl Acad Sci U S A.

[25] Heddi A, Nardon P (2005). *Sitophilus oryzae* L: a model for intracellular symbiosis in the Dryophthoridae weevils (Coleoptera). Symbiosis.

[26] Oakeson KF, Gil R, Clayton AL, Dunn DM, von Niederhausern AC, Hamil C, Aoyagi A, Duval B, Baca A, Silva FJ, Vallier A, Jackson DG, Latorre A, Weiss RB, Heddi A, Moya A, Dale C (2014). Genome degeneration and adaptation in a nascent stage of symbiosis. Genome Biol Evol.

[27] Fukatsu T, Koga R, Smith WA, Tanaka K, Nikoh N, Sasaki-Fukatsu K, Yoshizawa K, Dale C, Clayton DH (2007). Bacterial endosymbiont of the slender pigeon louse, *Columbicola columbae*, allied to endosymbionts of grain weevils and tsetse flies. Appl Environ Microbiol.

[28] Smith WA, Oakeson KF, Johnson KP, Reed DL, Carter T, Smith KL, Koga R, Fukatsu T, Clayton DH, Dale C (2013). Phylogenetic analysis of symbionts in feather-feeding lice of the genus *Columbicola*: evidence for repeated symbiont replacements. BMC Evol Biol.

[29] Nováková E, Hypša V (2007). A new *Sodalis* lineage from bloodsucking fly *Craterina melbae* (Diptera, Hippoboscoidea) originated independently of the tsetse flies symbiont *Sodalis glossinidius*. FEMS Microbiol Let.

[30] Chrudimský T, Husník F, Nováková E, Hypša V (2012). *Candidatus* Sodalis melophagi sp. nov.: phylogenetically independent comparative model to the tsetse fly symbiont *Sodalis glossinidius*. PLoS One.

[31] Koga R, Bennett GM, Cryan JR, Moran NA (2013). Evolutionary replacement of obligate symbionts in an ancient and diverse insect lineage. Environ Microbiol.

[32] Koga R, Moran NA (2014). Swapping symbionts in spittlebugs: evolutionary replacement of a reduced genome symbiont. ISME J.

[33] Gruwell ME, Hardy NB, Gullan PJ, Dittmar K (2010). Evolutionary relationships among primary endosymbionts of the mealybug subfamily Phenacoccinae (Hemiptera: Coccoidea: Pseudococcidae). Appl Environ Microbiol.

[34] Toju H, Hosokawa T, Koga R, Nikoh N, Meng XY, Kimura N, Fukatsu T (2010). “*Candidatus* Curculioniphilus buchneri”, a novel clade of bacterial endocellular symbionts from weevils of the genus *Curculio*. Appl Environ Microbiol.

[35] Toju H, Fukatsu T (2011). Diversity and infection prevalence of endosymbionts in natural populations of the chestnut weevil: relevance of local climate and host plants. Mol Ecol.

[36] Toju H, Tanabe AS, Notsu Y, Sota T, Fukatsu T (2013). Diversification of endosymbiosis: replacements, co-speciation and promiscuity of bacteriocyte symbionts in weevils. ISME J.

[37] Grünwald S, Pihofer M, Höll W (2010). Microbial associations in gut systems of wood- and bark-inhabiting longhorned beetles [Coleoptera: Cerambycidae]. Syst Appl Microbiol.

[38] Kaiwa N, Hosokawa T, Kikuchi Y, Nikoh N, Meng XY, Kimura N, Ito M, Fukatsu T (2010). Primary gut symbiont and secondary, *Sodalis*-allied symbiont in the scutellerid stinkbug *Cantao ocellatus*. Appl Environ Microbiol.

[39] Kaiwa N, Hosokawa T, Kikuchi Y, Nikoh N, Meng XY, Kimura N, Ito M, Fukatsu T (2011). Bacterial symbionts of the giant jewel stinkbug *Eucoryssus grandis* (Hemiptera: Scutelleridae). Zool Sci.

[40] Kaiwa N, Hosokawa T, Nikoh N, Tanahashi M, Moriyama M, Meng XY, Maeda T, Yamaguchi K, Shigenobu S, Ito M, Fukatsu T (2014). Symbiont-supplemented maternal investment underpinning host’s ecological adaptation. Cur Biol.

[41] Matsuura Y, Hosokawa T, Serracin M, Tulgetske GM, Miller TA, Fukatsu T (2014). Bacterial symbionts of a devastating coffee plant pest, the stinkbug *Antestiopsis thunbergii* (Hemiptera: Pentatomidae). Appl Environ Microbiol.

[42] Arp A, Munyaneza JE, Crosslin JM, Trumble J, Bextine B (2014). A global comparison of *Bactericera cockerelli* (Hemiptera: Triozidae) microbial communities. Environ Entomol.

[43] Dhami MK, Buckley TR, Beggs JR, Taylor MW (2013). Primary symbiont of the ancient scale insect family Coelostomidiidae exhibits strict cophylogenetic patterns. Symbiosis.

[44] Clayton AL, Oakeson KF, Gutin M, Pontes A, Dunn DM, von Niederhausern AC, Weiss RB, Fisher M, Dale C (2012). A novel human-infection-derived bacterium provides insights into the evolutionary origins of mutualistic insect–bacterial symbioses. PLoS Genet.

[45] Verbarg S, Frühling A, Cousin S, Brambilla E, Gronow S, Lünsdorf H, Stackebrandt E (2008). *Biostraticola tofi* gen. nov., spec. nov., a novel member of the family Enterobacteriaceae. Curr Microbiol.

[46] Werren JH, Windsor DM (2000). *Wolbachia* infection frequencies in insects: evidence of a global equilibrium?. Proc R Soc B.

[47] Hilgenboecker K, Hammerstein P, Schlattmann P, Telschow A, Werren JH (2008). How many species are infected with *Wolbachia*? – a statistical analysis of current data. FEMS Microbiol Let.

[48] Zug R, Hammerstein P (2012). Still a host of hosts for *Wolbachia*: analysis of recent data suggests that 40% of terrestrial arthropod species are infected. PLoS One.

[49] Perlman SJ, Hunter MS, Zchori-Fein E (2006). The emerging diversity of *Rickettsia*. Proc R Soc B.

[50] Weinert LA, Werren JH, Aebi A, Stone GN, Jiggins FM (2009). Evolution and diversity of *Rickettsia* bacteria. BMC Biol.

[51] Zchori-Fein E, Perlman SJ (2004). Distribution of the bacterial symbiont *Cardinium* in arthropods. Mol Ecol.

[52] Duron O, Bouchon D, Boutin S, Bellamy L, Zhou L, Engelstädter J, Hurst GD (2008). The diversity of reproductive parasites among arthropods: *Wolbachia* do not walk alone. BMC Biol.

[53] Nakamura Y, Kawai S, Yukuhiro F, Ito S, Gotoh T, Kisimoto R, Yanase T, Matsumoto Y, Kageyama D, Noda H (2009). Prevalence of *Cardinium* bacteria in planthoppers and spider mites and taxonomic revision of “*Candidatus* Cardinium hertigii” based on detection of a new *Cardinium* group from biting midges. Appl Environ Microbiol.

[54] Haselkorn TS, Markow TA, Moran NA (2009). Multiple introductions of the *Spiroplasma* bacterial endosymbiont into *Drosophila*. Mol Ecol.

[55] Nováková E, Hypša V, Moran NA (2009). *Arsenophonus*, an emerging clade of intracellular symbionts with a broad host distribution. BMC Microbiol.

[56] Fukatsu T (1999). Acetone preservation: a practical technique for molecular analysis. Mol Ecol.

[57] Fukatsu T, Nikoh N (1998). Two intracellular symbiotic bacteria of the mulberry psyllid *Anomoneura mori* (Insecta, Homoptera). Appl Environ Microbiol.

[58] Fukatsu T, Shibao H, Nikoh N, Aoki S (2001). Genetically distinct populations in an Asian soldier-producing aphid, *Pseudoregma bambucicola* (Homoptera: Aphididae), identified by DNA fingerprinting and molecular phylogenetic analysis. Mol Phylogenet Evol.

[59] Katoh K, Standley DM (2013). MAFFT multiple sequence alignment software version 7: improvements in performance and usability. Mol Biol Evol.

[60] Darriba D, Taboada GL, Doallo R, Posada D (2012). jModelTest 2: more models, new heuristics and parallel computing. Nat Methods.

[61] Guindon S, Gascuel O (2003). A simple, fast, and accurate algorithm to estimate large phylogenies by maximum likelihood. Syst Biol.

[62] Ronquist F, Teslenko M, van der Mark P, Ayres DL, Darling A, Höhna S, Larget B, Liu L, Suchard MA, Huelsenbeck JP (2012). MrBayes 3.2: efficient Bayesian phylogenetic inference and model choice across a large model space. Syst Biol.

[63] Stamatakis A (2006). RAxML-VI-HPC: maximum likelihood-based phylogenetic analyses with thousands of taxa and mixed models. Bioinformatics.

[64] Kikuchi Y, Fukatsu T (2003). Diversity of *Wolbachia* endosymbionts in heteropteran bugs. Appl Environ Microbiol.

[65] Matsuura Y, Kikuchi Y, Hosokawa T, Koga R, Meng XY, Kamagata Y, Nikoh N, Fukatsu T (2012). Evolution of symbiotic organs and endosymbionts in lygaeid stinkbugs. ISME J.

[66] Matsuura Y, Kikuchi Y, Meng XY, Koga R, Fukatsu T (2012). Novel clade of alphaproteobacterial endosymbionts associated with stinkbugs and other arthropods. Appl Environ Microbiol.

[67] Glasgow H (1914). The gastric caeca and the caecal bacteria of the Heteroptera. Biol Bull.

[68] Kikuchi Y, Hosokawa T, Fukatsu T, Dijk TV (2008). Diversity of bacterial symbiosis in stinkbugs. Microbial Ecology Research Trends.

[69] Abe Y, Mishiro K, Takanashi M (1995). Symbiont of brown-winged green bug, *Plautia stali* Scott. Jpn J Appl Entomol Zool.

[70] Fukatsu T, Hosokawa T (2002). Capsule-transmitted gut symbiotic bacterium of the Japanese common plataspid stinkbug, *Megacopta punctatissima*. Appl Environ Microbiol.

[71] Hosokawa T, Kikuchi Y, Nikoh N, Shimada M, Fukatsu T (2006). Strict host–symbiont cospeciation and reductive genome evolution in insect gut bacteria. PLoS Biol.

[72] Kikuchi Y, Hosokawa T, Fukatsu T (2007). Insect-microbe mutualism without vertical transmission: a stinkbug acquires beneficial gut symbiont from environment every generation. Appl Environ Microbiol.

[73] Kikuchi Y, Hosokawa T, Nikoh N, Meng XY, Kamagata Y, Fukatsu T (2009). Host–symbiont co-speciation and reductive genome evolution in gut symbiotic bacteria of acanthosomatid stinkbugs. BMC Biol.

[74] Prado SS, Almeida RP (2009). Role of symbiotic gut bacteria in the development of *Acrosternum hilare* and *Murgantia histrionica*. Entomol Exp Appl.

[75] Tada A, Kikuchi Y, Hosokawa T, Musolin DL, Fujisaki K, Fukatsu T (2011). Obligate association with gut bacterial symbiont in Japanese populations of southern green stinkbug *Nezara viridula* (Heteroptera: Pentatomidae). Appl Entomol Zool.

[76] Hosokawa T, Hironaka M, Mukai H, Inadomi K, Suzuki N, Fukatsu T (2012). Mothers never miss the moment: a fine-tuned mechanism for vertical symbiont transmission in a subsocial insect. Anim Behav.

[77] Hosokawa T, Hironaka M, Inadomi K, Mukai H, Nikoh N, Fukatsu T (2013). Diverse strategies for vertical symbiont transmission among subsocial stinkbugs. PLoS One.

[78] Salem H, Kreutzer E, Sudakaran S, Kaltenpoth M (2013). Actinobacteria as essential symbionts in firebugs and cotton stainers (Hemiptera, Pyrrhocoridae). Environ Microbiol.

[79] Kafil M, Bandani AR, Kaltenpoth M, Goldansaz SH, Alavi SM (2013). Role of symbiotic bacteria in the growth and development of the sunn pest *Eurygaster integriceps*. J Insect Sci.

[80] Taylor CM, Coffey PL, DeLay BD, Dively GP (2014). The importance of gut symbionts in the development of the brown marmorated stink bug, *Halyomorpha halys* (Stål). PLoS One.

